# Bioactive Compounds from *Pale Ale* Beer Powder Attenuate Experimental Colitis in BALB/c Mice

**DOI:** 10.3390/molecules27041194

**Published:** 2022-02-10

**Authors:** Paola D. D. S. Maia, Diego dos Santos Baião, Hayandra F. Nanini, Victor Paulo F. da Silva, Lissa Bantim Frambach, Iuri Matheus Cabral, Beatriz Pêgo, Beatriz E. Ribeiro, Mauro Sérgio Gonçalves Pavão, Vania M. F. Paschoalin, Heitor S. P. de Souza, Anna Paola T. R. Pierucci

**Affiliations:** 1 Basic and Experimental Nutrition Department, Josué de Castro Nutrition Institute, Federal University of Rio de Janeiro, Avenida Carlos Chagas Filho, 393, Rio de Janeiro 21941-590, Brazil; paolasmaia@hotmail.com (P.D.D.S.M.); victorpaulosf@gmail.com (V.P.F.d.S.); lissabantim@gmail.com (L.B.F.); iurimcabral@gmail.com (I.M.C.); appierucci@gmail.com (A.P.T.R.P.); 2 Institute of Chemistry, Federal University of Rio de Janeiro, Av. Athos da Silveira Ramos, 149, Rio de Janeiro 21941-909, Brazil; diegobaiao20@hotmail.com (D.d.S.B.); paschv@iq.ufrj.br (V.M.F.P.); 3 Department of Clinical Medicine, Clementino Fraga Filho University Hospital, Federal University of Rio de Janeiro, Rua Prof. Rodolpho Paulo Rocco 255, 11th floor, Rio de Janeiro 21941-617, Brazil; naninihf@gmail.com (H.F.N.); biapdamasceno@gmail.com (B.P.); bakerribeiro@gmail.com (B.E.R.); 4 Institute of Medical Biochemistry, Clementino Fraga Filho University Hospital, Federal University of Rio de Janeiro, Rua Prof. Rodolpho Paulo Rocco 255, 4th floor, Rio de Janeiro 21941-617, Brazil; pavaomsg@gmail.com; 5 D’Or Institute for Research and Education (IDOR), Rua Diniz Cordeiro 30, Botafogo, Rio de Janeiro 22281-100, Brazil

**Keywords:** bioactive compounds, experimental colitis, *Pale Ale* beer powder, phenolic acids

## Abstract

Phenolic compounds (PCs) present in foods are associated with a decreased risk of developing inflammatory diseases. The aim of this study was to extract and characterize PCs from craft beer powder and evaluate their potential benefits in an experimental model of inflammatory bowel disease (IBD). PCs were extracted and quantified from pure beer samples. BALB/c mice received either the beer phenolic extract (BPE) or beer powder fortified with phenolic extract (BPFPE) of PCs daily for 20 days by gavage. Colon samples were collected for histopathological and immunohistochemical analyses. Dextran sodium sulfate (DSS)-induced mice lost more weight, had reduced colon length, and developed more inflammatory changes compared with DSS-induced mice treated with either BPE or BPFPE. In addition, in DSS-induced mice, the densities of CD4- and CD11b-positive cells, apoptotic rates, and activation of NF-κB and p-ERK1/2 MAPK intracellular signaling pathways were higher in those treated with BPE and BPFPE than in those not treated. Pretreatment with the phenolic extract and BPFPE remarkably attenuated DSS-induced colitis. The protective effect of PCs supports further investigation and development of therapies for human IBD.

## 1. Introduction

Beer is an alcoholic drink produced by the brewing and fermentation of starches, mainly derived from malted barley, through the action of yeast, with the addition of hops. Special beers, also known as craft beers, have different characteristics compared with those produced on a large scale. The quality of these products is influenced by several variables, such as the quality of the raw material, malting type, wort preparation method, type of hops, type and quality of yeasts, fermentation time, maturation, and the use of processes such as pasteurization and filtration [1]. In addition to their worldwide acceptability, beers also have a growing appeal for functional properties because of the presence of antioxidant compounds, vitamin B complexes, and minerals [2]. The main class of antioxidant compounds found in beers is phenolic compounds (PCs), which are bountiful in malt and hops. Depending on the amount of each ingredient used for the manufacture of beer, it is estimated that approximately 70–80% of the PCs come from malt, whereas 20–30% come from hops [3]. PCs are abundantly distributed in foods of plant origin, such as fruits and vegetables [4]. In particular, beer has a variety of PCs, which directly contribute to various sensory characteristics of beer, and beer is the only significant source of hops with potential health-related benefits [2].

Research interest in PCs with antioxidant and anti-inflammatory properties has progressively increased in recent years, particularly because of their potential therapeutic benefits for chronic non-communicable diseases such as type 2 diabetes mellitus, hyperlipidemia, systemic arterial hypertension, cancer, and various inflammatory conditions [4,5,6,7,8]. Inflammatory bowel diseases (IBDs), including Crohn’s disease (CD) and ulcerative colitis (UC), are particularly interesting for the study of new anti-inflammatory strategies. Despite progress in elucidating the complex mechanisms underlying IBD, including genetic, immunologic, microbial, and environmental factors, the exact etiopathogenesis of IBD remains unclear [9,10]. Consequently, IBD remains incurable, and available therapies target immune mechanisms with limited and usually only temporary effectiveness [11,12]. In addition, the recent global increase in the incidence and prevalence of IBD, including in regions previously regarded as having low prevalence, has become an issue of major concern regarding public health [13,14].

Natural bioactive compounds, also classified as nutraceuticals or functional foods, have been tested in inflammatory and neoplastic disorders and have shown therapeutic properties in the gastrointestinal tract [15,16,17], particularly in mucosal healing [18,19]. Recently, increasing efforts have been directed toward the potential use of PCs for the treatment of IBD, in which they have been used as a complementary approach for medical therapy [20,21]. Currently, there is evidence supporting the relative efficacy of PCs in human IBD, particularly curcumin [22,23,24]. However, most data regarding the therapeutic actions of PCs in intestinal inflammation have been derived from in vitro and experimental studies in rodents. For instance, the beneficial effects of PCs have been reported in trinitrobenzene sulfonic acid (TNBS)-induced Wistar rats, showing a reduction in oxidative events and the expression of pro-inflammatory proteins attributed to the blockade of MAPKs and NF-κB signaling pathways [25]. 

The aim of this study was to determine the PCs in a microencapsulated Pale Ale craft beer powder and to investigate whether PCs extracted from Pale Ale craft beer powder have a potential therapeutic role in experimental colitis in mice.

## 2. Results

### 2.1. PCs Identification and Quantification

The total PC content in the beer powder fortified with phenolic extract (BPFPE) and beer phenolic extract (BPE) was 160.21 ± 2.17 and 123.16 ± 2.17 mg·g^−1^ of fresh weight basis (fwb), respectively (Table 1). Ten distinct PCs were identified and quantified in BPE and BPFPE: chlorogenic, di-OH-benzoic, ferulic, gallic, m-coumaric, p-coumaric, rosmarinic, syringic, vanillic, and 4-OH-phenylacetic acids. Considering only soluble phenolic extract, chlorogenic, di-OH-benzoic, gallic, vanillic, and 4-OH-phenylacetic were the most abundant PCs detected in the BPE and BPFPE. Gallic, rosmarinic, vanillic, and 4-OH-phenylacetic acids were found in both soluble and insoluble BPE and BPFPE. The content of chlorogenic, gallic, m-coumaric, rosmarinic, and acids found in the soluble phenolic extract of BPFPE was higher than that found in BPE; in the insoluble phenolic extract, the content of gallic and 4-OH-phenylacetic acid was higher in BPFPE than in BPE. Furthermore, gallic (soluble: 11.08 ± 0.07 + insoluble: 7.59 ± 0.01 mg·g^−1^ of fwb), rosmarinic (soluble: 6.61 ± 0.08 + insoluble: 7.44 ± 1.01 mg·g^−1^ of fwb), vanillic (soluble: 11.82 ± 4.05 + insoluble: 8.32 ± 1.19 mg·g^−1^ of fwb), and 4-OH-phenylacetic (soluble: 9.21 ± 1.89 + insoluble: 7.52 ± 0.99 mg·g^−1^ of fwb) acids were the most abundant PCs detected in the BPE. Similar results were obtained for the BPFPE. 

### 2.2. Dextran Sodium Sulfate (DSS) Induction Leads to Clinical and Anatomic Signs of Inflammatory Activity 

**Figure 1:**
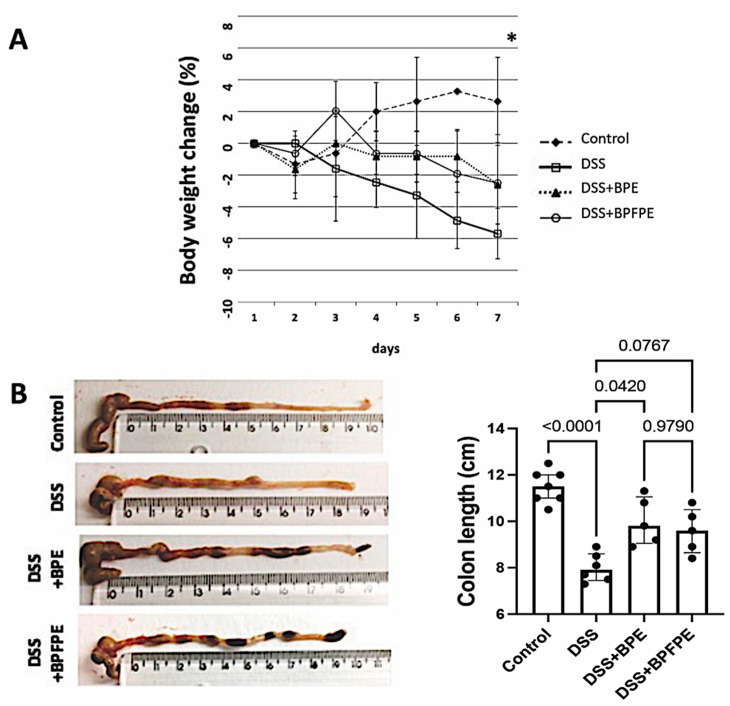
Effect of pretreatment with BPE and the BPFPE on the clinical and anatomical parameters of the colitis model. Following dextran sodium sulfate (DSS)-induction, animals presented a progressive weight loss compared with control animals on day 7 (* *p* = 0.007) (**A**). Colon length shortening following DSS-induction was significantly attenuated upon pretreatment with BPE and BPFPE (**B**). Values are medians with interquartile ranges of two independent experiments, with 2 to 4 animals per group. The analysis was performed by Brown–Forsythe and Welch analysis of variance (ANOVA) tests in which multiple comparisons were performed using the Dunnett’s T3 test. Significant values are presented.

To study the possible anti-inflammatory activity of beer PCs, wild C57Bl/6 mice received either BPE or BPFPE daily for 20 days by gavage. After treatment, the mice were induced to develop acute colitis with 2.5% DSS in drinking water for seven days. We assessed the post-induction body weight changes daily. Mice in the control group had a cumulative weight gain from day four onwards. Mice in groups exposed to DSS, however, demonstrated a tendency toward a decrease in body weight compared with the control group. However, on day seven, only the untreated mice exposed to DSS differed from the control mice (*p* = 0.007) (Figure 1A). Next, we measured the colon length after euthanasia. The DSS-induced group exhibited a significantly shortened colon compared with the control group. Colon length was preserved in the BPE-treated animals (Figure 1B).

### 2.3. BPE and BPFPE Attenuate Microscopic Inflammation in DSS-Induced Acute Colitis 

**Figure 2:**
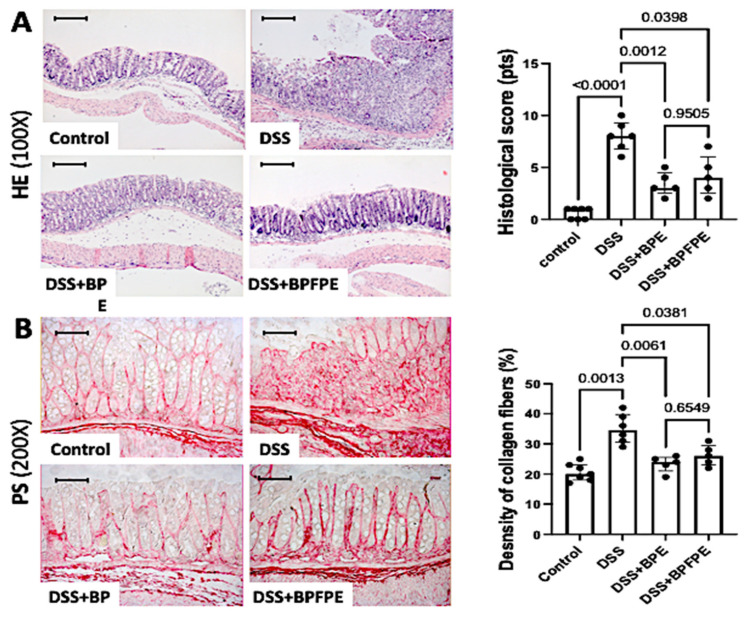
Effect of pretreatment with BPE and BPFPE on the histological parameters and the deposition of collagen fibers in the colon. Paraffin sections stained with hematoxylin and eosin (HE) were analyzed according to predefined histological parameters, and sections with phosphomolybdic acid-picrosirius red dye (PS) were quantified by a computerized image analysis system. Animals treated with BPE or BPFPE exhibited significantly less inflammation (**A**) and lower density of collagen fibers (**B**) compared with DSS-induced animals. Values are medians with interquartile ranges of two independent experiments, with 2 to 4 animals per group. The scale bars represent 50 μm. The analysis was performed by Brown–Forsythe and Welch ANOVA tests in which multiple comparisons were performed using the Dunnett’s T3 test. Significant values are presented.

The distal colon samples were removed for histopathological analysis. Colitis-induced mice displayed severe disruption of colonic integrity, including mucosal erosions and ulcerations, and a dense inflammatory infiltrate in the lamina propria, when compared with controls. Treatment with either BPE or BPFPE markedly prevented the development of complete inflammatory changes and damage induced by DSS exposure. Both BPE- and BPFPE-pretreated DSS-induced mice had lower histological scores than the DSS-induced group (Figure 2A).

Collagen deposition was significantly higher in the colons of mice subjected to DSS than in the colons of control mice. However, collagen deposition was significantly prevented in BPE- and BPFPE-pretreated mice compared with DSS-induced mice (Figure 2B).

### 2.4. Pretreatment with BPE and BPFPE Decreases the Loss of Goblet Cells and Apoptotic Rates in the Colon of DSS-Induced Mice 

**Figure 3:**
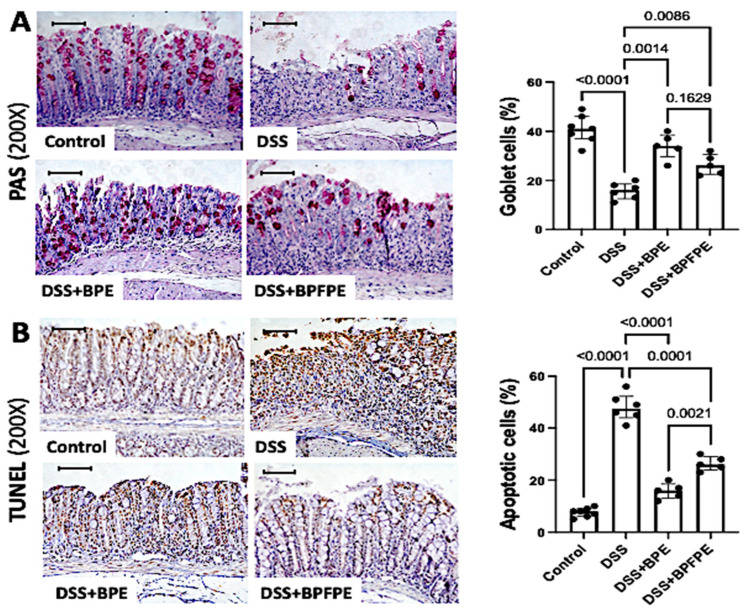
Effect of pretreatment with BPE and BPFPE on the density of goblet cells and on apoptotic rates within the colon. Paraffin sections are stained with periodic acid-Schiff (PAS), and, using the terminal deoxynucleotidyl transferase (TdT)-mediated dUTP nick-end labeling (TUNEL) assay for detecting apoptotic cells, were analyzed using a computerized image analysis system. Animals induced with DSS presented a remarkable loss of goblet cells (**A**) and a dramatic increase in apoptotic rates (**B**) in colon samples, significantly prevented by the administration of BPE and BPFPE. Values are medians with interquartile ranges of two independent experiments, with 2 to 4 animals per group. The scale bars represent 50 μm. The analysis was performed by Brown–Forsythe and Welch ANOVA tests in which multiple comparisons were performed using the Dunnett’s T3 test. Significant values are presented.

Tissue damage resulting from DSS-induced colitis remarkably reduced the presence of goblet cells within the colonic mucosa. Pretreatment with BPE or BPFPE significantly attenuated goblet cell loss compared with that in DSS-induced mice (Figure 3A). Colon apoptosis was assessed using the in situ terminal deoxynucleotidyl transferase (TdT)-mediated dUTP nick-end labeling (TUNEL) assay. The apoptotic rate was increased in the colon of DSS-induced mice compared with controls but was significantly attenuated in both BPE- and BPFPE-pretreated groups (Figure 3B). 

### 2.5. Pretreatment with BPE and BPFPE Decreases the Accumulation of Immune Cells in the Colonic Lamina Propria of DSS-Induced Mice

**Figure 4:**
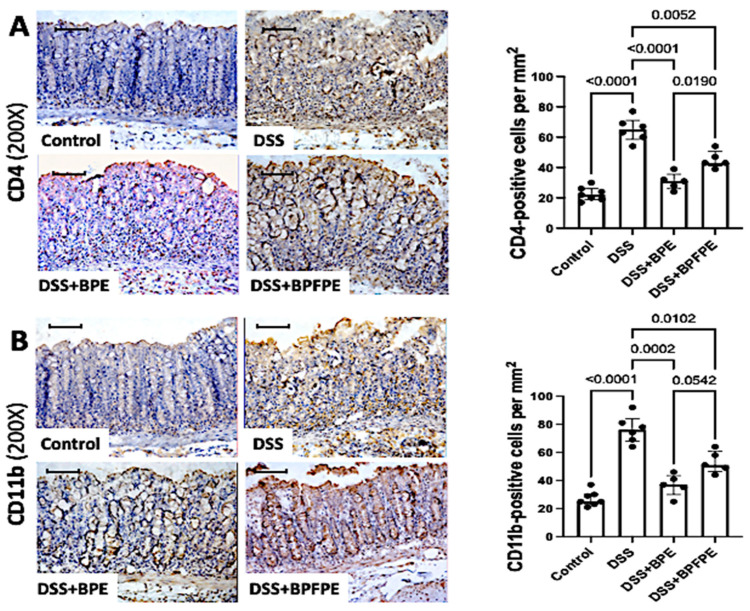
Effect of pretreatment with BPE and the BPFPE on the CD4- and CD11b-positive cell infiltration within the colon. Paraffin sections stained with indirect immunoperoxidase were analyzed using a computerized image analysis system. Animals treated with BPE and BPFPE exhibited significantly fewer CD4-positive T cells (**A**) and macrophages (CD11b-positive cells) (**B**) compared with vehicle-treated DSS-induced animals. Values are medians with interquartile ranges of two independent experiments, with 2 to 4 animals per group. The scale bars represent 50 μm. The analysis was performed by Brown–Forsythe and Welch ANOVA tests in which multiple comparisons were performed using the Dunnett’s T3 test. Significant values are presented.

Next, we performed immunohistochemistry to label CD4- and CD11b-cells (Figure 4A) to further characterize the different inflammatory cell populations present in the lamina propria. Similar results were found for the CD4-positive and CD11b-positive cell populations. BPE- and BPFPE-pretreated mice accumulated fewer CD4- and CD11b-positive cells in the lamina propria compared with DSS-induced mice (Figure 4B). 

### 2.6. Pretreatment with BPE and BPFPE Blunts NF-κB and ERK Activation

**Figure 5:**
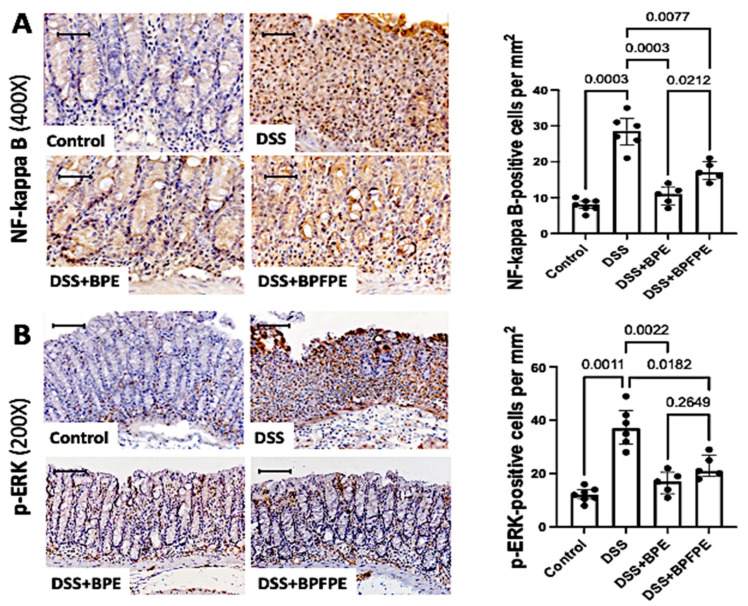
Effect of pretreatment with BPE and the BPFPE on the expression of intracellular signaling pathways involved in the inflammatory response triggered by DSS-induction. Paraffin sections stained with indirect immunoperoxidase were analyzed using a computerized image analysis system. Animals treated with BPE and BPFPE exhibited significantly less NF-κB (**A**) and fewer phosphorylated ERK-positive cells (**B**) compared with those of DSS-induced animals. Values are medians with interquartile ranges of two independent experiments, with 2 to 4 animals per group. The scale bars represent 50 μm. The analysis was performed by Brown–Forsythe and Welch ANOVA tests in which multiple comparisons were performed using the Dunnett’s T3 test. Significant values are presented.

To further analyze the potential anti-inflammatory activity of the PCs, the NF-κB and ERK intracellular signaling pathways were investigated by immunohistochemistry (Figure 5A). DSS-induced mice displayed a higher density of NF-κB and phosphorylated-ERK protein expression in the epithelium and lamina propria of the colon compared with control mice. NF-κB and p-ERK expression was significantly attenuated in mice pretreated with BPE and BPFPE compared with mice exposed to DSS only (Figure 5B). 

## 3. Discussion

In the present study, we characterized for the first time the PCs present in a Pale Ale craft beer powder obtained from a new method of extraction and investigated whether a microencapsulated preparation of PCs could have a therapeutic action in an experimental model of IBD in mice. Notably, the procedure proposed in this study resulted in the successful extraction of several PCs, which proved to be beneficial in preventing the inflammatory process and tissue damage resulting from DSS-induced experimental colitis.

Currently, providing a product that, apart from being rich in PCs, is attractive in appearance, flavor, and practicality and adequate in serving size, continues to represent a major challenge. In addition, it is critically important to delay the loss of bioactive compounds and browning of the product. Microencapsulation, the technique used in the current study, stands out among the various technologies used for the development of functional products because of its ability to concentrate and stabilize important bioactive compounds, making it an attractive alternative for the production of new food products [26]. The most nutritionally and economically viable method for the production of microparticles is atomization because it can reduce volume and mass by removing water and alcohol, thus stabilizing and concentrating nutrients and bioactive substances in food products. In addition, similar to the results of our study, this method maintains the stability of components susceptible to oxidation and heat, along with the sensory characteristics of general appearance, color, and taste [27].

In this study, we used powdered beer and phenolic extracts with an organic solvent to extract soluble PCs, along with serial extractions with acid and alkaline solvents to obtain insoluble PCs before identification and quantification by high-performance liquid chromatography (HPLC). After performing the extraction processes, ten PCs were identified and quantified in the beer sample (chlorogenic, di-OH-benzoic, ferulic, gallic, m-coumaric, p-coumaric, rosmarinic, syringic, vanillic, and 4-OH-phenylacetic acids), where gallic, rosmarinic, vanillic, and 4-OH-phenylacetic were found in larger quantities in both products because they were present in both soluble and insoluble extracts. Previous studies analyzing beer samples yielded distinct results. For instance, Zhao et al. (2010) [28] analyzed thirty-four beer samples, including twenty-seven local and seven imported from the city of Guangdong, China, by HPLC-coupled to mass spectrometry. Nine PCs were identified: gallic, protocatechin, catechin, vanillic, caffeic, syringic, epicatechin, p-coumaric, and ferulic. In a study by Moura-Nunes et al. (2016) [29], ten PCs from Brazilian commercial beers were identified by HPLC: 3,4-dihydroxybenzoic, 3,4-dihydroxyphenylacetic, 4-hydroxyphenylacetic, 5-caffeoylquinic, benzoic, p-coumaric, ferulic, gallic, syringic, and vanillic acids. Nevertheless, the current study revealed a different pattern of extracted compounds. Although we found PCs, including chlorogenic, m-coumaric, and rosmarinic acids, we did not identify other compounds, such as protocatechuic, catechins, epicatechin, and 5-caffeoylquinic acids. Thus, as with PC content, different strains, raw materials, and fermentation processes likely influenced the phenolic profile during beer production [30]. Moreover, soluble PCs are found in vacuoles, whereas insoluble compounds are covalently bound to structural components of the cell wall of foods, such as cellulose, hemicellulose, lignin, pectin, and structural proteins [31]. Therefore, this insoluble fraction could not be extracted by organic solvents and was released only after alkaline and acid treatments. Although alkaline hydrolysis breaks the ester bonds, acid hydrolysis breaks the glycosidic bonds, generally leaving the ester bonds intact [31]. 

In recent years, these bioactive compounds have attracted the interest of the scientific community and the food industry because high consumption of foods rich in PCs has been associated with lower rates of chronic diseases such as diabetes and cardiovascular and inflammatory diseases [31]. In a multicenter prospective cohort study conducted in Europe, dietary polyphenol intake was measured to investigate its potential association with disease development during the follow-up period. Although significance was established only for flavones and resveratrol, most polyphenols were associated with decreased odds of developing CD [32]. Further, several studies have investigated the potential direct therapeutic benefits of PCs in IBD. Although current data on PCs for treating IBD do not exhibit consistent effectiveness [33], several studies using curcumin for both UC and CD have generated promising results [23,34]. Several natural PCs have been known to exert beneficial effects against various diseases, in addition to their intrinsic antioxidant and scavenging properties [35,36]. Currently, the rationale for the potential protective effects of PCs is based not only on their antioxidant actions but also on other anti-inflammatory and immunomodulatory properties [37,38]. Therefore, this study aimed to investigate the possible effects of PCs on other well-established mechanisms involved in DSS-induced colitis. Consequently, we verified that all typical clinical, morphological, and histological abnormalities observed in DSS-induced colitis were clearly attenuated by pretreatment with either BPE or BPFPE. 

In particular, the in vivo model used in this study showed that tissue damage and epithelial cell loss from apoptosis were markedly attenuated, corroborating previous in vitro studies showing a protective role of PCs, where cell viability and expression levels of tight junction proteins increased [39]. Another important element resulting from intestinal inflammation, the deposition of collagen fibers, was also assessed in this study, exhibiting an important attenuation with pretreatment with BPE or BPFPE. In agreement with this protective effect, a previous study using another chemically induced colitis in mice demonstrated the suppressive action of a PC in intestinal fibrosis through the inhibition of the reactive oxygen species-dependent TGF-beta 1/SMADs pathway [40]. Nevertheless, not all beneficial effects can be attributed solely to antioxidant actions, and some PCs exhibit activity similar to non-steroidal anti-inflammatory drugs without common adverse effects related to the gastrointestinal tract [41]. For instance, the attenuation in mononuclear cell accumulation in the intestinal lamina propria observed in DSS-induced mice pretreated with PCs is likely reflecting indirect effects on cell migration. It is possible that the increased presence and accumulation of activated CD4-positive T cells and CD11b-postive cells in the intestinal mucosa, an expected response to local injury, may be affected by the downregulation of proinflammatory mediators, including cytokines and chemokines [42,43,44]. 

Some PCs have been shown to inhibit the expression of genes related to proinflammatory mediators and modulate the signaling of transcription factors in inflammatory pathways and antioxidants [45]. In different experimental models of colitis, PCs have exhibited anti-inflammatory actions, usually through interference with NF-κB, resulting in the downregulation of several inflammatory mediators, including the suppression of downstream pro-inflammatory cytokines [46,47]. NF-κB has been recognized as a key pro-inflammatory transcription factor, and p65 is typically involved in the inflammatory response [48]. In this study, the results confirmed the clear attenuation of NF-κB expression and nuclear translocation, based on the immunolabelling of the p65 subunit, in the colonic mucosa of mice pretreated with BPE and BPFPE. In contrast, the NF-κB pathway is known to be induced by stressful stimuli, including free radicals, proinflammatory cytokines, and microbe-associated molecular patterns (MAMPs) [48]. Hence, it is possible that, in addition to direct inhibition of NF-κB activation, PCs may also affect the gut microbiota, acting as prebiotics, promoting the integrity of the intestinal mucosa [49], while at the same time boosting the antioxidant response [50]. In addition to these potential actions, it has been proposed that inhibition of NF-κB by PCs might occur through the inhibition of IkK phosphorylation and/or the prevention of proteasomal degradation of IkB [51,52]. Nevertheless, we also detected a remarkable attenuation of p-ERK expression in mice pretreated with BPE and BPFPE. The effects of polyphenols on ERK have been previously identified in vitro in a study of human mast cells isolated from the intestinal mucosa, which showed that resveratrol inhibited the phosphorylation of ERK [53]. Among the few studies focusing on MAPK-related effects following polyphenol treatment, raspberry polyphenols were shown to reduce the activation of ERK1/2 MAPKs, prevent IkK degradation, and inhibit nuclear translocation of the p65 subunit of NF-κB [25]. Altogether, this evidence appears to corroborate the existence of multiple redundant pathways underlying the inflammatory process of IBD and experimental colitis and reinforce the beneficial effects of PCs, potentially acting at different and complementary levels within the inflammatory network.

Although the current study presents a successful methodological procedure for the extraction of PCs from beer powder, which showed relevant biological actions in experimental colitis, important limitations should be acknowledged. First, this study did not investigate the exact mechanisms by which PCs exert their protective actions in the model. The number of experiments and animals per experiment was relatively small, particularly owing to technical difficulties regarding the care of animals, follow-up, and use of the oral route in daily administration. Therefore, although we recognize that the findings of this study should be interpreted with caution, the overall results were consistent and showed significant differences. Hence, the successful data generated in this study appear to serve, at least in part, as a guide for future investigations. Further studies on the subject should also consider other dosages and routes of administration, different animal models, and protocols for analyzing prophylactic versus therapeutic applications. In addition, characterizing the functional activities of specific PCs would help identify compounds with the most beneficial biological actions. Next, the separation and purification of the most active PCs would likely enhance their therapeutic effects. Finally, analyzing more specific protective mechanisms related to intestinal inflammation will need to consider the role of the gut microbiome and its potential modulation by PCs. In fact, it has been shown that polyphenols may modulate the balance of microbiota by acting as prebiotics in humans [54] and in rodents [55,56]. It is currently believed that PCs can promote the diversity of gut microbiota, thereby directly affecting the maintenance of intestinal homeostasis and health [57].

In conclusion, specific bioactive PCs extracted from craft beer samples were effectively delivered orally in a microencapsulated preparation and prevented the complete development of experimental colitis in mice induced with DSS. Pretreatment with BPE and BPFPE remarkably attenuated DSS-induced colitis in mice by preserving mucosal integrity, resulting in less inflammatory cell infiltration based on the activation of the NF-κB and ERK signaling pathways. The protective effect of PCs extracted from powdered craft beer supports the idea of further investigation and development of therapies for human IBD based on dietary interventions.

## 4. Methods

### 4.1. Powdered Pale Ale Craft Beer

*Pale Ale* craft beer was prepared as previously described [58]. The production process was developed at the Institute of Microbiology by Professor Paulo de Goes/UFRJ and the raw materials such as *Pale Ale* malt, *Hersbrucker* hops, *Nottingham* yeast, 600 mL amber glass bottles for storage, and metal stoppers (Pry-off) to close the bottles were acquired at the Rio de Janeiro (Brazil) local market. 

The powdered *Pale Ale* craft beer was prepared without matrix encapsulation by spray-drying process according to [59], using a Mini Spray Dryer Büchi model B-290 (Büchi Laboratoriums Technik, Flawil, Switzerland) with some modifications. Three operating conditions of inlet air temperature of 180 °C, outlet temperature variable from 80 to 100 °C (depending on the inlet temperature), and a sample feed flow of 17 mL·min^−1^ and a 0.3 mm diameter nozzle were used. The feed solution was stored under magnetic stirring and pumped into the main drying chamber using a peristaltic pump, with an aspiration rate of 32 m^3^·h^−1^, compressor air pressure of 0.03 MPa, and feed flow rate of 0.36 L·h^−1^. The particles were produced in triplicate, and the powdered products were stored in desiccators at −80 °C.

### 4.2. Phenolic Extract Production

The extraction of soluble PCs from powdered *Pale Ale* craft beer was performed according to the adapted methodology as in Inada et al. (2015) [60]. Soluble PCs were extracted using cold ethanol: in a deionized and distilled water solution (H_2_O-DD) (80:20, *v*/*v*), stirred for 10 min, and centrifuged at 2500× *g* at 10 °C for 5 min. This step was performed twice, the supernatants were combined, the solvents were removed using a rotary evaporator at 130 rpm, and the dry residues were reconstituted with drinking water. The extract was stored at −80 °C until it was used to fortify the beer powder and offer it to the mice.

### 4.3. PCs Contents

The PC analysis of isolated phenolic extract and powdered *Pale Ale* craft beer fortified with phenolic extract was performed according to a methodology adapted from Baião et al. (2017) [61] using soluble and conjugate phenolic extraction methods. Soluble PCs were extracted according to the method described in Section 4.2. *Phenolic Extract Production*. The dry residue was reconstituted in H_2_O-DD.

Conjugated PCs were extracted via alkaline and acid hydrolysis. Alkaline hydrolysis was performed in 10 M NaOH, and H_2_O-DD was added to the residues after soluble PC extraction. The residue was stirred at 130 rpm for 16 h at room temperature to prevent light exposure. The residue was adjusted to pH 2 with concentrated HCl and extracted with ethyl acetate. After centrifugation at 2500× *g* for 5 min at 10 °C, the supernatants were collected, and the alkaline hydrolysis process was repeated twice. The supernatants were combined, the solvents were removed using a rotary evaporator at a speed of 130 rpm, and the dried residues were reconstituted in methanol: H_2_O-DD (80:20 *v*/*v*).

Acid hydrolysis was performed by adding 100% HCl to the residues following alkaline extraction. The residues were stored in a water bath at 85 °C for 30 min, and extraction with ethyl acetate was performed. After centrifugation at 2500× *g* for 5 min at 10 °C, the supernatants were collected and the acid hydrolysis process was repeated two more times. All extracts were filtered through 0.45 µm cellulose ester membranes (Merck Millipore Co., Darmstadt, Germany) prior to HPLC.

The HPLC device was equipped with a 5 µm reverse phase C18 column (250 × 4.6 mm, I.D., Ascentis^®^, Los Angeles, CA, USA) guarded by a 5 µm C18 guard column (10 × 3.0 mm, I.D., Ascentis^®^) and a diode-array detector (DAD) detector model SPD-M30A (Shimadzu, Kyoto, Japan). The DAD wavelength was monitored from 190 to 370 nm. The column temperature was set at 40 °C, and the injection volume was 20 µL for all samples. The mobile phase (1.0 mL·min^−1^) was 0.3% formic acid (in H_2_O-DD), methanol (100%), and acetonitrile (100%) with gradient elution. The column was equilibrated using 81% formic acid, 18% methanol, and 1% acetonitrile. After sample injection, formic acid and methanol concentrations increased to 79% and 20% in 1 min, 56% and 43% in 18 min, and 14% and 85% in 23 min, respectively, and remained constant for up to 30 min. Between the injections, the column was re-equilibrated with 81% formic acid, 18% methanol, and 1% acetonitrile for 10 min. PC quantification was performed using a calibration curve (1–50 ppm) with standards at a minimum of five concentrations. Reference standards for phenolic compounds used in this study were purchased from Fluka Analytical (Buchs, Switzerland) and Sigma-Aldrich (St. Louis, MO, USA). All standards were of analytical grade, with a minimum of 95% purity and were used as received.

### 4.4. In Vivo Bioactivity Assessment of Phenolic Extract and Powdered Pale Ale Craft Beer

To assess bioactivity in vivo, phenolic extract or powdered *Pale Ale* craft beer fortified with phenolic extract was tested in isolation in mice with induced colitis.

#### Mice

The Institutional Animal Care Committee of the Health Sciences Centre of the Federal University of Rio de Janeiro approved the care and use of the animals and procedures reported in this study (approval ID: 01200.001568/2013-87), in accordance with the guidelines of the International Care and Use Committee of the National Institutes of Health and the Guide for the Care and Use of Laboratory Animals (National Research Council, 2011). Male BALB/c mice (*Mus musculus*), 6- to 8-weeks-old and 25–30 g, originating from the vivarium located at Naval Marcílio Dias Hospital (HNMD)**,** were maintained under specific pathogen-free conditions, constant temperature (22 °C), and 12/12 h light and dark cycles in the vivarium of the Nutrition Department (CCS/UFRJ). The mice were provided with a standard autoclaved platelet formula and filtered water ad libitum during the experimental period, and were dewormed 15 days before starting the experiment with a 0.2 mg dose of the Drontal dewormer applied directly to the mouth of each animal with a Pasteur pipette. Subsequently, mice were randomly assigned to one of four groups of two to four animals each (in two experiments).

### 4.5. Experimental Design

Each treatment group was housed in the same rack-mounted wire cage with sufficient space to accommodate all mice and reduce potential heterogeneities in the gut microbiota composition [62]. During the entire experiment, clinical manifestations such as diarrhea, bleeding, and weight changes were recorded for all animals in the study. The four groups into which animals were randomly assigned were: group I (n = 7), without supplementation and induction of colitis (control); group II (n = 6), without supplementation, only induction of colitis (with oral dextran sodium sulfate, DSS); group III (n = 5), supplemented with 800 μL of the beer phenolic extract (DSS + BPE); and group IV (n = 5), supplemented with 0.6 g pure beer powder diluted in 600 μL of phenolic extract, beer powder fortified with phenolic extract (DSS + BPFPE). Different amounts of each intervention were administered depending on the solid content and the PCs in the solutions, resulting in the same amount of PCs (Table 1). Aliquots of phenolic extract and BPFPE stored at −80 °C were removed 30 min before the start of supplementation, along with the water and feed of the cages of the animals subjected to the procedure, such that gastric emptying would occur. The supplement was administered to the animals for 20 days by intragastric gavage.

On day 21, the mice were induced with a seven-day experimental acute colitis with 2.5% DSS salt (DSS, 36,000–50,000 MW, MP Bioscience) in the drinking water, according to Boussenna et al. [63]. The control group received neither PCs nor DSS in the drinking water. Body weight and food ingestion were assessed daily during the induction of colitis. Water consumption was monitored and found to be similar between groups. On day 28, the animals were euthanized by asphyxiation using CO_2_ gas, followed by confirmation of death through cervical dislocation. The distal colon samples were removed and measured. 

### 4.6. Histological Sample Preparation and Evaluation

After the abdominal cavity inventory, the entire colon was carefully removed. Colon length was measured, and the distal 2 cm was immediately divided into portions to perform the experimental procedures. Colon samples were fixed in 40 g/L formaldehyde saline, embedded in paraffin, and cut into 5 μm sections. The slides were stained with hematoxylin and eosin (HE) and examined microscopically by two independent observers. HE staining was used for qualitative evaluation of inflammatory changes. Inflammation was assessed based on three parameters, with specific scores attributed to each: (1) epithelial changes (prolonged epithelial cell or crypt = 1 pt, barrier disruption = 2 pts, ulceration < 50% of the area = 3 pts, ulceration > 50% of the area = 4 pts); (2) immune cell infiltration (mild = 1 pt, moderate = 2 pts, severe = 3 pts); and (3) submucosal immune cell infiltration (mild = 1 pt, moderate = 2 pts, severe = 3 pts) [64].

### 4.7. Goblet Cell and Collagen Deposition Assessment in the Colon 

To further analyze histopathological changes, we used the periodic acid-Schiff (PAS) technique to stain goblet cells within the intestinal epithelium. The density of the goblet cells was defined as the percentage of PAS-positive cells within at least 500 epithelial cells in the crypts and the surface epithelium of longitudinally sectioned intestinal pits. To assess the deposition of collagen fibers within the colon, sections were stained with phosphomolybdic acid picrosirius red (PS) dye. At least 10 different areas per tissue section were analyzed using light microscopy. The density of collagen fibers was defined as the area positively stained for collagen in relation to the total intestinal tissue using an imaging analysis system.

### 4.8. Immunohistochemistry 

Distal colon sections were first incubated at 90 °C in 0.01 M sodium citrate buffer (pH 6.0) for 30 min for antigen retrieval. The slides were then immersed in 3% hydrogen peroxide for 10 min to block endogenous peroxidase activity. After rinsing in phosphate-buffered saline (PBS), the tissue sections were incubated with 1.5% bovine serum albumin and 0.1% Triton for 30 min and subsequently with 5% bovine serum albumin for 10 min. Staining was performed using the following primary antibodies: rabbit monoclonal anti-CD4 antibody (1:50; Santa Cruz Biotechnology Inc., Santa Cruz, CA, USA), rabbit monoclonal anti-CD11b antibody (1:100; ab133357, Abcam, Cambridge, UK), mouse monoclonal anti-p65 to NF-κB (1:100) (Santa Cruz Biotechnology, USA), and mouse monoclonal anti P-ERK1/2 phosphorylated ERK (1:100) (Santa Cruz Biotechnology, USA). Two sections from each sample were incubated with PBS alone and served as the negative control. After incubation in a humidified chamber overnight at 4 °C, the tissue sections were rinsed with PBS and incubated with MACH 4 Universal HRP-Polymer (Biocare Medical, LLC, Pacheco, CA, USA) for 30 min at room temperature. The development was performed using a hydrogen peroxide and diaminobenzidine (Dako, Glostrup, Denmark) solution. The samples were counterstained with Harris’s hematoxylin, dehydrated, and mounted onto slides using the water-free mounting medium Entellan^®^ (Merck Millipore, Darmstadt, Germany). 

### 4.9. Assessment of Apoptosis in the Colon

Apoptosis was assessed in histological sections using the TUNEL assay. Colonic tissue was analyzed using an ApopTag peroxidase in situ apoptosis detection kit (Millipore Corporation, Billerica, MA, USA). First, the paraffin sections were deparaffinized, hydrated, and incubated with proteinase K solution. Endogenous peroxidase activity was blocked by immersion in 3% hydrogen peroxide for 10 min. The slides were rinsed in PBS and incubated with a solution containing the TdT enzyme. The second section from each sample was incubated without the TdT enzyme to serve as a negative control. For positive controls, samples were pretreated with DNase I (Sigma-Aldrich, Deisenhofen, Germany). Following the TdT enzyme step, tissues were incubated with an anti-digoxigenin antibody peroxidase conjugate. The sections were developed using a solution containing hydrogen peroxide and diaminobenzidine, counterstained with Harris’s hematoxylin, dehydrated, and mounted in a mounting medium. Apoptotic cells were defined as morphologically preserved TUNEL-positive cells and apoptotic bodies.

### 4.10. Quantitative Assessment of Colon Sections

All histomorphological analyses of the tissue sections under light microscopy were performed by two experienced independent observers who were unaware of the experimental data. Quantitative analysis was performed using a computer-assisted image analyzer (Leica QWin Plus V 3.5.1, Leica Microsystems Ltd., Heerbrugg, Switzerland). Any epithelial and lamina propria cells exhibiting an identifiable reactivity distinct from the background were regarded as positive. The results of the quantitative analysis of the cell subsets are expressed as the number of cells per mm^2^ of longitudinally sectioned colon tissues. 

### 4.11. Statistical Analysis

The PC contents of the BPFPE and BPE were compared using two-way analysis of variance (ANOVA) followed by Bonferroni post hoc correction. Results were considered significant at *p* < 0.05. Statistical differences between the experimental groups were evaluated using the Wilcoxon matched-pairs signed rank test or Brown–Forsythe and Welch (ANOVA) tests, in which multiple comparisons were performed using Dunnett’s T3 test, as appropriate. Values are expressed as medians with interquartile ranges. All tests were two-tailed, and the statistical significance was set at *p* < 0.05. Values are expressed as mean ± standard deviation (SD), and statistical analyses were performed using Prisma software version 9.1.2 for Windows (GraphPad Software, San Diego, CA, USA).

## Figures and Tables

**Table 1:** Phenolic compounds content in beer powder fortified with phenolic extract (BPFPE) and beer phenolic extract (BPE).

	BPFPE (mg·g^−1^ fwb)	BPE (mg·g^−1^ fwb)
***Soluble Phenolic Compounds***		
Chlorogenic acid	16.04 ± 0.08 ^a,^*	12.28 ± 1.08 ^a^
Di-OH-benzoic acid	14.81 ± 1.12 ^b^	11.39 ± 2.82 ^a,b^
Ferulic acid	10.52 ± 0.20 ^c^	8.09 ± 2.20 ^b^
Gallic acid	14.41 ± 0.01 ^b,^*	11.08 ± 1.07 ^a,b^
*m*-Coumaric	12.11 ± 2.09 ^c,^*	9.32 ± 1.59 ^b^
*p*-Coumaric	6.67 ± 1.37 ^e^	5.12 ± 2.37 ^c^
Rosmarinic acid	8.59 ± 0.68 ^d,e,^*	6.61 ± 0.58 ^c^
Syringic acid	9.58 ± 1.12 ^c,d^	7.37 ± 2.12 ^b,c^
Vanillic acid	15.37 ± 1.05 ^a,b^	11.82 ± 1.05 ^a^
4-OH-phenylacetic acid	11.97 ± 1.69 ^b,c^	9.21 ± 1.89 ^a,b^
***Insoluble phenolic compounds***		
Gallic acid	9.87 ± 0.21 ^a,^*	7.59 ± 0.11 ^a^
Rosmarinic acid	9.67 ± 1.01 ^a^	7.44 ± 1.51 ^a^
Vanillic acid	10.82 ± 1.19 ^a^	8.32 ± 1.39 ^a^
4-OH-phenylacetic acid	9.78 ± 0.99 ^a,^*	7.52 ± 0.29 ^a^
Total	160.21 ± 2.17 *	123.16 ± 2.17

Values are expressed as mean ± standard deviation for triplicate analysis. Different letters within the same column for each beer product indicate statistically significant differences between the phenolic compounds at a significance level of *p* < 0.05. * denotes the difference from BPE at a significance level *of p* < 0.01.

## Data Availability

Materials such as protocols, analytic methods, and study material are available upon request to interested researchers. The raw data supporting the conclusions of this manuscript will be made available by the authors, without undue reservation, to any qualified researcher.

## Sample Availability

Samples from the compounds used in this study are stored at −80 °C and are available upon request to any qualified researcher.
